# MicroRNA expression profile of human advanced coronary atherosclerotic plaques

**DOI:** 10.1038/s41598-018-25690-4

**Published:** 2018-05-18

**Authors:** Mariana S. Parahuleva, Christoph Lipps, Behnoush Parviz, Hans Hölschermann, Bernhard Schieffer, Rainer Schulz, Gerhild Euler

**Affiliations:** 1 Internal Medicine/Cardiology and Angiology, University Hospital of Giessen and Marburg, Location Marburg, Germany; 2 Internal Medicine I/Cardiology and Angiology, University Hospital of Giessen and Marburg, Location Giessen, Germany; 3 Krankenhaus Bad Homburg Innere Medizin I – Kardiologie, Bad Homburg, Germany; 4 Institute of Physiology, Justus Liebig University, Giessen, Germany

## Abstract

MicroRNA (miR) is reported to be involved in vascular inflammation and may represent a novel class of diagnostic biomarkers in cardiovascular disease. We aimed to identify the miR expression profile in human advanced coronary atherosclerotic plaques (CAP) and to connect this expression to the processes in atherosclerosis. Microarray techniques and TaqMan polymerase chain reaction were used to analyse the global expression of 352 miRs in CAP obtained during ACS MULTI-LINK study. 11 miRs were selected on the basis of their implication in atherosclerosis, endothelial activation, and inflammation. 6 miRs were found to be differently expressed in CAP when compared to non-atherosclerotic internal mammary arteries (IMA, p < 0.05). The expression of miR-21, -92a, and -99a was verified and found to be significantly up-regulated in CAP versus IMA (p < 0.001). We also performed bioinformatic analysis and found several potential target genes of miR-92a and -99a as well as several pathways with impact on atherosclerosis which could be differently expressed due to this miRNA profile. The most up-regulated miRs are involved in processes known to be connected to atherosclerosis. Interfering with the miR expression in the artery wall is a potential way to affect atherosclerotic plaque and cardiovascular disease development.

## Introduction

Monocytes/macrophages play important roles in the formation of atherosclerotic lesions^1,2^. In addition, an inflammatory response takes place, and circulating monocytes migrate into the intima, where they differentiate to macrophages^3^. The presence of different growth factors induces the formation of atherosclerotic lesions and when they reduce coronary vessel lumen significantly and/or the plaque is complicated by a thrombus, acute myocardial infarction (AMI) may occur^4^. Advanced vulnerable plaques are rich in inflammatory cells, mostly only ‘classically polarized’ macrophages, and are highly susceptible to rupture^4,5^. These plaques represent a high risk particularly with the standard invasive diagnosis by coronary angiography. The plaque rupture is considered as the most important mechanism underlying most acute ischemic syndromes, including acute coronary syndrome (ACS) and stroke. Although the pathophysiology of plaque rupture is not completely understood, it is now well accepted that lesion vulnerability is more closely correlated to plaque composition than size. As a new regulatory layer, several microRNAs (miRs) have been found to modulate the function of endothelial cells (ECs), smooth muscle cells (SMCs) and macrophages by controlling the expression levels of chemokines and thereby affecting different stages in the progression of atherosclerosis^6–8^. MiRs play critical roles in the cardiovascular biology through regulating more than one third of human genes by binding to the 3′untranslated region of target gene mRNAs^6^. Because miRs are upstream regulators of gene expression and are involved in various physiological and pathological processes, it would be needful to address their role in vascular inflammation and vessel remodelling, in particular in leukocyte activation and their infiltration into the vascular wall^9,10^. MiRs have been also widely shown to have diagnostic and therapeutic value in vascular inflammatory diseases and vascular cell damage^11–13^. Most investigations on miRs in human atherosclerotic plaques have been done in stroke patients. Cipollone *et al*. were one of the first groups identifying 4 miRs that are specially expressed in symptomatic plaques from stroke patients^14^. These miRs were confirmed in an independent study by Maitrias *et al*.^15^. In the meantime some more studies in humans and animal studies have been done, all focussing on involvement of miRs in carotid-related stroke (Maitrias *et al*.^16^. Besides the importance of miRs in stroke, miRs are aberrantly expressed in patients with ACS, and specific circulating as well as cellular miRs have been shown to be associated with the clinical subtype of ACS and could be used as biomarker^11,12^. However, no information is currently available with respect to cellular miRs expression and their modulation in human coronary atherosclerotic lesions under inflammatory condition during the early phase of AMI development *in vivo* and this will be the object of this case-control study. We identified abundantly expressed miRs in advanced coronary plaques from patients with ACS with microarray techniques and further analyzed selected miRNA candidates by qRT-PCR. Furthermore, we evaluated miRNA expression pattern in human coronary atherosclerotic specimens from patients who had sustained an AMI compared to healthy control arteries (A. mammaria interna). Our observations will identify plaques miRNA candidates, which are correlated with early acute phase of myocardial infarction and vascular inflammation and may help to develop future diagnostic and therapeutic abilities to identify and evaluate cardiovascular disease and particular ACS.

## Material and Methods

### Ethics Statement

The protocol for the study and all experimental protocols were approved by the Ethics Committee of the University of Giessen and informed written consent was obtained from all subjects. Furthermore, all methods were carried out in accordance with relevant guidelines and regulations.

### Study population and study design

This study is monocentric, case-control trial and included 12 patients with acute coronary syndrome (ACS) during ACS MULTI-LINK study admitted to the Department of Internal Medicine I Cardiology/Angiology, University Hospital of Giessen-Marburg^17^. Age, gender, BMI (body mass index), blood pressure levels, history of previous myocardial infarction, cardiovascular risk factors including systematic hypertension, diabetes mellitus, smoking, hyperlipidemia, and family history of coronary artery disease (CAD) will be assessed and recorded at study entry (Table 1). In addition to the diseased coronary samples 14 unaffected internal mammary arteries (IMA) were obtained during coronary bypass surgery and were used as control group.

**Table 1:** Study population and characteristics of lesion.

№	Age/Gender	BMI (kg/gm)	Risk factors	Type of stenosis	Location of lesion	Number of sections per plaque	Macrophages (CD68) in the shoulder region
1	80/W	22.43	H	deNovo	RCx	1	++
2	73/W	25.16	H	deNovo	RCA	3	+
3	73/M	25.26	H	deNovo	LAD	2	++
4	54/M	27.78	F	deNovo	LAD	2	++
5	68/M	33.02	O	deNovo	LAD	1	+
6	76/M	33.17	DM II, H, O	restenosis	LAD	1	+
7	62/M	30.61	DM II, H, O	deNovo	LAD	2	++
8	61/M	27.76	H, O	restenosis	LAD	3	+
9	65/M	27.14	N, O	deNovo	LAD	1	++
10	65/M	28.08	H, O, N	deNovo	LAD	3	+
11	41/W	18.52	H, DM L, F	deNovo	LAD	3	++
12	47/M	30.30	H, O	deNovo	LAD	1	++

LAD, left anterior descending; RCx, Ramus circumflexus; RCA, right coronary artery; BMI, Body-Mass-Index, DM, diabetes mellitus typ I and II; H, hypertension; L, hyperlipoproteinaemia; F, family disposition; N, nicotine; O, obesity; M, man; W, woman.

0 = no detectable staining; + = weak positive staining; ++ = strong positive staining.

### Tissue samples

Atherectomy lesions were obtained from 12 patients with symptomatic coronary artery disease who underwent percutaneous directional coronary atherectomy (DCA) according to standard techniques^18^. 14 unaffected IMA were obtained during coronary bypass surgery and were used as healthy controls. All tissue samples were immediately frozen in liquid nitrogen and stored at −80 °C until use. The specimens were embedded in OCT compound and snap-frozen. Tissue samples were cut into ~20 μm thick cryostat sections (LeicaCM 1900, LeicaMicrosystems, Wetzlar, Germany) at a temperature of −22 °C and stored in 2 ml Eppendorf-tubes.

### Histological and immunohistochemical analyses

Hematoxylin-eosin stained sections from each OCT block were examined to establish the morphological characteristics of the plaques, in accordance with the classification of Stary^19^.

### RNA/miRNA-Isolation

Total RNA was extracted from 23 tissue sections obtained from 12 coronary atherosclerotic plaques (Table 1) using the Roti-Quick-Kit (Carl Roth GmbH, Karlsruhe, Germany) following the manufacturer’s instructions and as described previously^10,12^. RNA concentrations were measured using an Eppendorf BioPhotometer. In addition, we used the SABiosciences RT² qPCR-Grade miRNA Isolation Kit (SABiosciences Corporation, Frederick, MD, USA) to enrich miRNA from 40 µg of total RNA of each sample according to manufacturer’s instructions. This kit combines a phenol/chloroform-based extraction method with a silica membrane spin column technology. MiRNA was then reverse transcribed using the SABiosciences RT² miRNA FirstStrand Kit according to the manufacturer’s protocol and as described previously^10,12^.

### MiRNA expression profile

Samples were analyzed with the SABiosciences Human miFinder RT² microRNA PCR Array, 96-well (SABiosciences Corporation, Frederick, MD, USA) according to the manufacturer’s guidelines and as described previously^10,12^. Briefly, simply mix cDNA template, generated from the first strand kit, with the appropriate ready-to-use PCR master mix. For PCR array analysis, aliquots of the mixture were placed in each well of a 96-well RT2 miRNA profiler miFinder PCR array plate that contained a panel of primer sets for a thoroughly researched set of 88 pathway- or disease-focused miRNAs (miRs), plus four small nuclear RNA housekeeping (SNORD 44, 47, 48, and RNU6-2) assays. The relative amount of each miR in PCR array analysis was normalized to an average of four small nuclear housekeeping genes. Applying the NormFinder algorithm for calculation of stability values SNORD 44 was identified as best housekeeping gene and used for normalisation. One sample per group was analyzed using four different miRNA PCR array-plates, a total of 352 miRs, to identify miRs which are robustly expressed across the groups and profoundly different between patients with ACS and healthy controls. Array-results were evaluated by www.sabiosciences.com/mirna_pcr_assay_search.php. The initial array simply represents a kind of decision guidance together with the current literature about miRs as potential biomarkers for cardiovascular disease for the selection of candidates which were further investigated by real-time RT-PCR and does not have any quantitative value. A flow chart of this study can be found as Supplementary Figure S1. The study consisted of three general parts: a systematic literature review for selecting candidate miRs, an in silico analyse phase for screening candidate miRs, and a validation phase for confirming optimal miRs. For the literature review, we preliminarily selected candidate miRs from published studies based on the following inclusion criteria: diagnostic potential of miRs as biomarkers for coronary plaque rupture and extracellular communicators in cardiovascular disease confirmed by at least 2 publications. Then, we excluded unqualified candidates according to the following exclusion criteria: (1) miRs detected mostly in non-cardiac tissue or non-cardiovascular patients; (2) obvious differences in methodology; (3) miRs detected in small sample size (n ≤ 30). Next, the miRs, which were differentially regulated in the array and have predicted targets related to cardiovascular pathogenesis and well-documented role in plaque growth and stability identified via TargetScan, miRBa-se.org and microRNA.org, were screened in the in silico analysis phase (see Supplementary Table S1). In the validation phase, the optimal miRs as potential marker for coronary plaques instability and rupture *in vivo* were examined using real-time RT-PCR.

### Real-time reverse transcriptase polymerase chain reaction (real-time RT-PCR)

Relative miRNA quantification of specific miRs in tissue samples was performed by real-time RT-PCR using RT² SYBR Green qPCR Master Mix (SABiosciences Corporation, Frederick, MD, USA) and CFX 96 real-time system Bio-Rad (Bio Rad, Munich, Germany) according to the manufacturer’s protocol. Briefly, a total of 20 µl were added to each well, containing 1 µl of reverse transcribed miRNA, 1 µl of miRNA qPCR Assay primer, 8 µl of ddH_2_O and 10 µl of master mix per well. Thermal protocol contained 10 min of denaturation at 95 °C followed by 40 cycles of 95 °C for 15 sec, 55 °C for 40 sec and 72 °C for 30 sec for hybridization and elongation. Real-time RT-PCR reactions were performed in triplicates. MiRs were considered as present when CT-values (threshold cycle) were lower than 30. SNORD 44 was used as housekeeping gene for normalisation. Gene expression was assessed using the 2−ΔΔCt calculation method as described previously^10,12^.

### MircoRNA target prediction

We performed bioinformatic sequence analysis of miR-92 and -99, which showed differential expression between the advanced atherosclerotic plaques and control group in qRT-PCR validation. The microRNA databases and target prediction tools miRBase (http://microrna.sanger.ac.uk/), PicTar (http://pictar.mdc-berlin.de/) and TargetScan (http://www.targetscan.org/index.html) were used to identify potential microRNA targets. Specifically, we searched for targets with known expression in cardiovascular tissue with a special focus on predicted interactions with molecules of interest (VCAM, ICAM, eNOS, heparan sulfate, CD68, and CD40) and signalling pathways (inflammatory, ischaemic and endothelial activation pathways, such as mTOR or NFkappaB). We focused on targets predicted by at least two prediction data bases and containing a miR-92-8mer or miR-99-8mer seed match in the respective 3′UTR region^7,20^.

### Data presentation and statistical analysis

Data are presented as box plot with median (25th/75th percentiles) and whiskers (Tukey). Data were found to be not normally distributed according to D’Agostino & Pearson omnibus normality test and were compared using Kruskal-Wallis test followed by Dunn’s corrections for multiple comparisons. The alpha value was 0.05 and the adjusted p-values are presented in each graph. All statistical calculations were performed using the statistical package GraphPad Prism, version 6.05 (GraphPad Software, Inc., USA). All hypotheses were 2-tailed, and a probability value of less than 0.05 was considered statistically significant.

## Results

### Study population and plaques characteristics

To assess the role of miRNAs (miRs) in coronary atherosclerosis, we compared atherosclerotic lesion formation from patients with acute coronary syndrome (ACS). A collective of 23 sections from 12 coronary lesions (10 primary and 2 restenosis lesions) obtained from 12 patients with ACS were investigated (Table 1). Comparison of cardiovascular risk factors revealed a higher prevalence only for hypertension and obesity, but not for diabetes and hypolipoproteinaemia (Table 1). Semiquantitative data from pathological examination and immunostaining with antibodies against CD68 (a macrophage antigen) of advanced coronary atherosclerotic plaques, classified as Stary types IV–V, are summarized in Table 1.

### Selection strategy of relevant miRs related to advanced coronary atherosclerotic plaques

To determine the influence of advanced atherosclerosis on the levels of miRNA expression in coronary plaques, we performed initially a miRNA profile using miRNA PCR array covering 352 human miRs. Based on the microarray data we focused on miRs, which were robustly expressed across all investigated groups and profoundly different between patients with acute coronary syndrome (ACS) and healthy controls. In addition, we performed a systematic literature review and selected miRs with diagnostic potential as biomarkers for coronary plaque rupture and extracellular communicators in cardiovascular disease that were detected in patients with coronary artery disease (CAD) (see Supplementary Fig. S1). We first excluded miRs detected in non-cardiac tissues or in patients with non-cardiovascular disease (n = 235). We then excluded controversial miRs which were investigated by divergent methodologies (n = 11) and studies which enrolled fewer than 30 samples (n = 23). Thus, a total of 88 miRs were finally selected for screening in the in silico analysis phase. In this phase, further array was performed to compare the expression of candidate miRs between coronary artery plaques (CAP, n = 4) and unaffected internal mammary artery (IMA) as healthy controls (n = 4). No significant differences were observed between CAP and IMA in the other 49 miRs. In silico analysis of miRs (n = 39) was then performed by database screening (TargetScan.org, miRBa-se.org, microRNA.org) to determine relevant miRNA biomarker candidates according to their predicted interactions with molecules of interest, and conduction of careful in silico analysis, studying the literature for relevant miRs associated with vascular inflammation and atherosclerosis, cardiovascular pathogenesis, including endothelial injury, endothelial activation, and vascular inflammation^21^, as well as the impact of their target genes on plaque growth and stability was considered. Following this analysis, we identified 11 miRs of interest for vascular and plaque inflammation (see Supplementary Table S1).

Multiple of these miRs were significantly downregulated in CAP compared with unaffected IMA (p < 0.05; Table 2). Although the absolute number of significantly downregulated miRs prevailed, some plaques miRs were identified demonstrating increased levels (Table 2). Interestingly, most if not all of the highly expressed and significantly upregulated miRs in advanced coronary plaques are known to be expressed in the vascular wall, particularly in endothelial cells. These endothelial miRs include miR-92a, miR-21, miR-29, and members of the let-7 family (let-7f) (Table 2). In contrast to the high levels and profound upregulation of endothelial and vascular miRs, cardiac- and smooth muscle-expressed miRs were detected particularly at low levels (Table 2). miRs related with inflammation (miR-155, miR-181b) were not profoundly regulated in advanced coronary plaques. Finally, from a miRNA profiling in a matched derivation case-control cohort, 11 miRs were carried over to the validation phase and were chosen for further investigation using real-time RT-PCR (Table 2).

**Table 2:** Up- and Down-regulated miRs in advanced coronary atherosclerotic plaques (CAP versus non-atherosclerotic a. mammaria samples (IMA).

miRNA	IMA		CAP		Fold change	P-values
	Mean	SD	Mean	SD		
let-7f	−0.217	0.834	−4.954	1.384	−4.7	0.001
miR-1	−0.559	3.772	−5.654	1.104	−4.7	0.001
miR-9	−3.307	1.793	−4.146	1.298	−2.4	0.05
miR-19b	−0.079	1.375	3.998	1.321	2.7	0.05
miR-21	−0.106	2.026	5.685	1.289	4.5	0.001
miR-22	−0.183	0.997	−4.980	2.323	−4.5	0.05
miR-29b	−1.098	1.287	4.011	1.165	2.8	0.05
miR-92a	−1.061	1.505	3.768	1.322	2.6	0.05
miR-99a	−1.061	1.334	4.979	1.278	3.8	0.001
miR-143	−0.188	1.777	−3.090	1.384	−2.7	0.05
miR-223	−0.172	1.397	3.694	1.537	3.5	0.05

miRs were detected with the SABiosciences Human miFinder RT² microRNA PCR Array in non-atherosclerotic a. mammaria samples (IMA, n = 4) or advanced coronary atherosclerotic plaques (CAP, n = 4). Relative mRNA quantification was performed and the fold change in the target miR, normalized to the internal control (SNORD 44) and relative to the expression in healthy controls, was calculated and presented (cut off >2). Significance was assumed at p < 0.05 (corrected p-values**)**.

### Differentially regulated miRs in advanced coronary plaques

**Figure 1:**
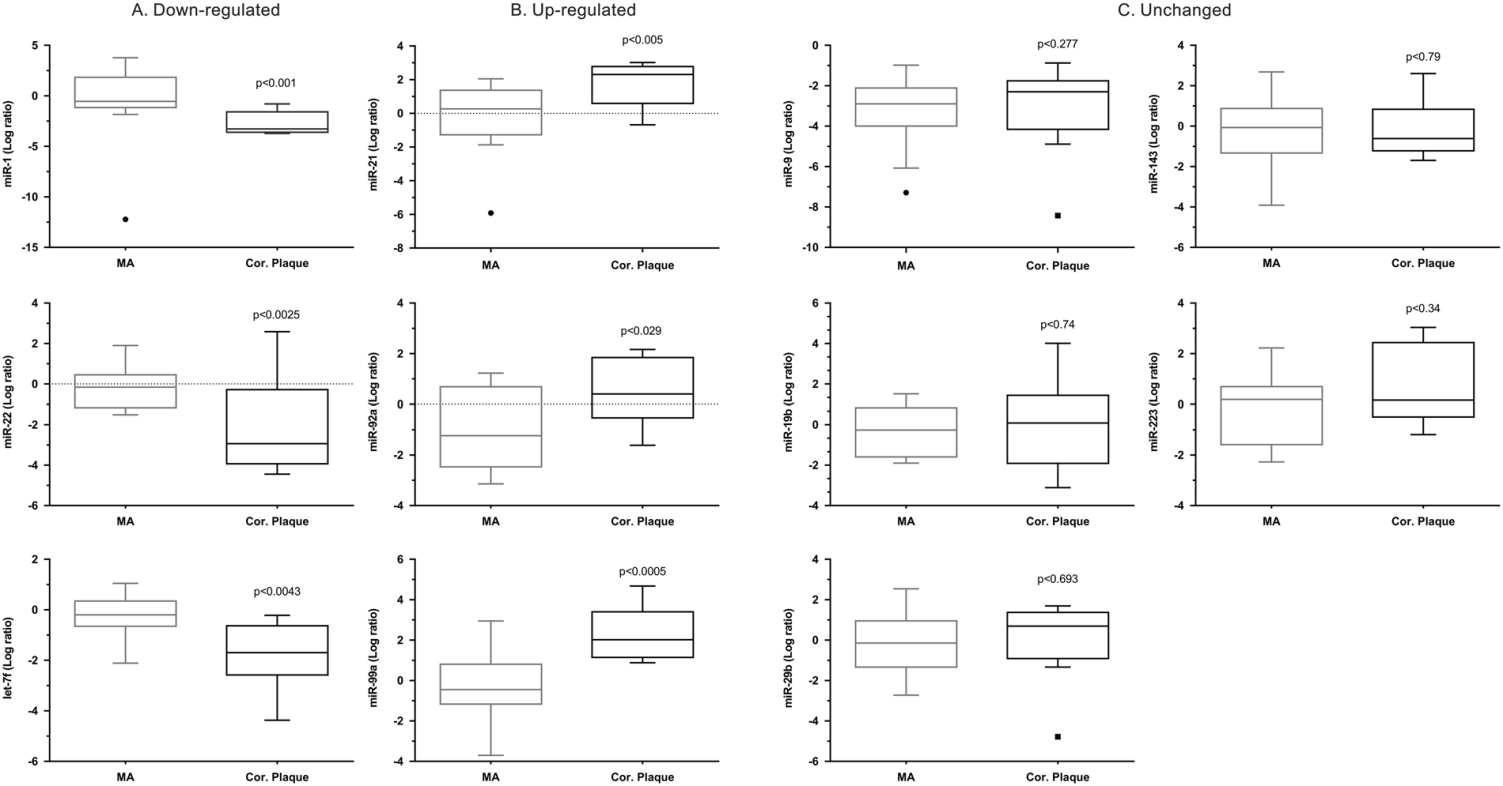
RT-qPCR-analysis of expression of miRs in human coronary atherosclerotic plaques (CAP) from patients with acute coronary syndromes (ACS) (n = 12), who underwent percutaneous directional coronary atherectomy (DCA) and from unaffected specimen from the internal mammary artery (IMA) as a control group (n = 14): (**A**) miRs down-regulated expression in CAP vs. control group (IMA), (**B**) miRs up-regulate expression in CAP vs. control group (IMA), and (**C**) miR whose expression did not alter. Snord44 was used as reference gene for normalization and the relative miRNA expression was calculated using the 2^−ΔΔCT^ method. Data are presented as box plot with median (25th/75th percentiles) log ratios (Tukey) and compared using Kruskal-Wallis test followed by Dunn’s corrections for multiple comparisons. The alpha value was 0.05 and the adjusted p-values compared to control group are presented in each graph.

Based on the microarray data, 11 miRs: let7f, miR-1, miR-19b, miR-9, miR-21, miR-29a, miR-29b, miR-92a, miR-99a, miR-143, and miR-223, which are linked to cardiovascular function/regeneration were selected for further analysis in advanced atherosclerotic plaques obtained from coronary arteries from patients (n = 12) with ACS and compared with non-atherosclerotic IMA samples (n = 14). Expression profiles of the miRs were verified using TaqMan real-time qPCR. As shown in Fig. 1, according to their expression pattern across the patient’s samples the investigated miRs could be classified into 3 different groups, (A) down-regulated expression vs. control, (B) up-regulated expression vs. control and (C) unchanged expression. Out of 11 miRs, 3 miRs were reliably detected and significantly up-regulated: miR-21, miR-92a, and miR-99a while the expression of miR-1, miR-22, and let-7f was down-regulated (Fig. 1).

### Target gene prediction and verification

**Figure 2:**
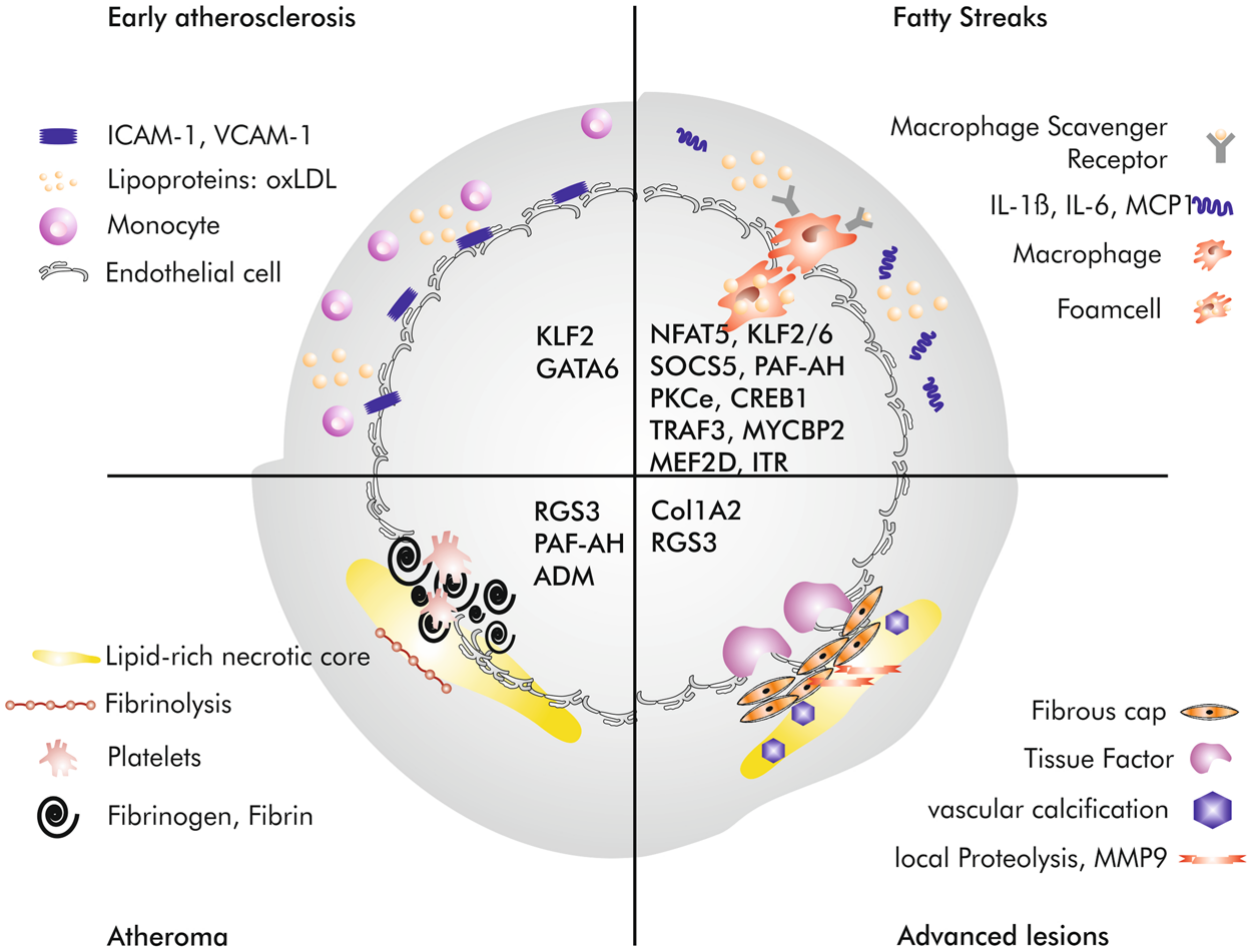
Molecular imaging of target genes of miR-92a at different stages of atherosclerotic plaque progression. Schematic cross section of coronary artery demonstrating different phase of atherosclerotic lesion progress: from early atherosclerosis to advanced plaques. The examples of appropriate target gene of miR-92a at each stage of plaque progression are listed in black in the middle of the figure.

Our search for predicted target genes of miR-92a and -99a found 308 and 32 potential targets, respectively, using TargetScan PicTar database (see Supplementary Tables S2 and S3). We then performed a database screening (TargetScan.org, miRBa-se.org, microRNA.org) to determine relevant miRNA biomarker candidates according to their predicted interactions with molecules of interest (VCAM, ICAM, eNOS, heparan sulfate, CD68, and CD40) and signalling pathways (inflammatory, ischaemic and endothelial activation pathways, such as mTOR or NFkappaB). Interestingly, many of the miR-92a target genes are related to atherosclerosis. These potential target genes of miR-92a as well as several pathways with impact on atherosclerosis are listed in Table 3 and depicted in Fig. 2.

**Table 3:** Predicted miR-92a target genes and their impact on atherosclerosis via PicTar.

Gene symbol	Gene Name	Impact on Atherosclerosis	Reference (inter alia)
USF2	Upstream Transcription Factor 2	related to familial hypercholsteremia	Chen *et al*., 2014, *Int J Mol Med*.
MEF2D	Myocyte Enhancer Factor 2D	regulates proliferation of VSMCs	Zhao *et al*., 2002, *Arch Biochem Biophys*.
GDF11	Growth Differentiation Factor 11	protects against endothelial cell injury	Mei *et al*., 2016, *Mol Ther*.
RGS3	Regulator of G-protein Signalling 3	protects against pathological changes of adventitial fibrobloasts	Xu *et al*., 2017, *Cell Biochem Funct*.
KLF2	Kruppel-Like Factor 2	modulated endothelial homeostasis, vasoregulation, vascular growth/remodeling, and inflammation	Novodvorsky *et al*., 2014, *Prog Mol Biol Transl Sci*.
NADPH Oxidase	NicotinAmidadenindinucleotidPhosphat Oxidase	impact on vascular oxidative stress	Di Pietro *et al*., 2017, *Int J Mol Sci*.
TRAF3	Tumor necrosis factor Receptor-Associated Factor 3	modulates CD40 signaling in atherogenesis	Zirlik *et al*., 2007, *Arterioscler Thromb Vasc Biol*.
CD51	CD51	expressed on endothelial microparticles	Arteaga *et al*., 2006, *Am J Cardiol*.
GRK5	G protein-coupled Receptor Kinase-5	attenuates atherosclerosis by regulating receptor tyrosine kinases	Wu *et al*., 2012, *Arterioscler Thromb Vasc Biol*.
Adrenomedullin	adrenomedullin	marker of carotid plaques	Gottsäter *et al*., 2013, *J Hypertens*.
KLF4	Kruppel-Like Factor 2	promotes transition of VSMC phenotype	Shankman *et al*., 2015, *Nat Med*.
MYCBP2	Myc Binding Protein 2	suppresses M2-like phenotypes in macrophages	Pierre *et al*., 2017, *Eur J Immunol*.
RYR3	Ryanodine Receptor 3	RYR3 gene polymorphisms associates with atherosclerosis	Zhao *et al*., 2014, *BMC Cardiovasc Disord*.
CREB1	Cyclic adenosine monophosphate Response Element-Binding protein	enhances interleukin-17A production and inflammation	Kotla *et al*., 2013, *Sci Signal*.
COL1A2	Collagen 1A2	enhanced expression in human aortal intima during atherogenesis	Shchelkunova *et al*., 2013, *Biochemistry (Mosc)*.
GATA6	GATA binding protein 6	regulates adehsion moleculaes in endothlial cells	Tsoyi *et al*., 2010, *Atherosclerosis*.
NFAT5	Nuclear Factor of Activated T-cells 5	drives macrophage migration	Halterman *et al*., 2012, *Front Physiol*.
ITR	Inotocin Receptor	enhances VSMC hyperplasia	Kang *et al*., 2015, *PLoS One*.
PKCε	Protein Kinase C epsilon	inflammation and smooth muscle cell dysfunction	Raghuraman *et al*., 2016, *Atherosclerosis*.
PAF-AH	Platelet-Activating Factor AcetylHydrolase	modulation of inflammation and plaque formation	Karabina *et al*., 2010, *Biochimie*

## Discussion

The main cause of cardiovascular disease in patients with atherosclerosis is myocardial infarction (MI) and stroke^22^. The rupture of atherosclerotic plaques is the major cause of mortality and morbidity of atherosclerosis in humans^23^. To minimize the extent of atherosclerosis after a severe or recurrent MI or stroke, therapeutic strategies are needed to limit atherosclerotic lesion size in the early phase of plaque growth and to prevent rupture of instable plaques. The traditional treatment for MI such as medical therapy, percutaneous coronary intervention (PCI), and coronary artery bypass grafting can reduce myocardial ischemia, improve cardiac function, and lower the risk of sudden death, but not fundamentally solve the problem with atherogenesis. Moreover, recent data from the ORBITA study have shown that in patients with medically treated stable angina and anatomically and haemodynamically severe coronary stenosis, PCI did not increase exercise time by more than the effect of a placebo procedure^24^.

The involvement of miRNAs (miRs) in cell-to-cell communication and modulation of inflammatory processes during physiological and pathological conditions such as atherosclerosis suggests their use as novel therapeutic tool in the early phase of plaque growth^9,25^. Moreover, we have identified recently the human circulating monocytes as putative biomarkers and as novel carriers for the cell-specific transfer of miRs in the early phase of MI^12^. For a better understanding of the underlying pathophysiology of plaque growth and rupture, particularly for the development of drugs, which can ultimately prevent MI or stroke, the present study was set up to investigate, for the first time, the expression of 352 miRs and their predicted targets in human advanced coronary atherosclerotic plaques (CAP). Of the studied miRs, we confirmed that the expression of three-i.e., miR-21, -92a, and -99a-is highly up-regulated in atherosclerotic plaques, which led us to predict that these miRs were related to the regulation of several genes involved in key processes of atherosclerosis. Moreover, the expression of miR-21 was up-regulated in symptomatic compare to asymptomatic carotid plaques whereas the expression of miR-92a and -99a was not regulated^10^. Thus, our data present significant evidence that plaque miRNA represent a potential atherosclerosis marker and has unique expression profiling in different arterial regions: miR-21 is a specific miRNA marker of human symptomatic carotid atherosclerotic plaques whereas miR-92a belongs to miRNA profiling of human advanced CAP. Furthermore, miR-21 can be used as a predictive indicator for vascular restenosis of lower extremity arterial occlusive disease after interventional therapy and compared with bare-metal stents, anti-21 – coated stents effectively reduced in-stent restenosis in ballon-injured human IMA^26,27^.

Emerging evidence indicates that alteration of flow conditions regulate expression of miRs in endothelial cells (ECs) both *in vitro* and *in vivo*. These flow-sensitive miRs, known as “mechanosensitive-miRs”, regulate endothelial gene expression, and can regulate endothelial dysfunction and atherosclerosis^28,29^. Interestingly, miR-21 belongs to mechanosensitive miRs with pro- and anti-atherogenic effect, whereas miR-92a is a mechanosensitive athero-miR and was shown to induce endothelial dysfunction and pro-atherogenic responses^30–32^. In the present study, we show, that both mechanosensitive athero-miR-21 and -92a are significantly up-regulated in human advanced CAP versus IMA (fold changes 4.5 (p < 0.005) and 2.6 (p < 0.029), respectively). Furthermore, we demonstrate that miR-92a is expressed in human coronary atherosclerotic lesions, more frequent in left anterior descending artery (LAD, Table 1), where the endothelial shear stress is higher compared to the Ramus circumflexus (RCx) and right coronary artery (RCA). The effort to explain this preferential susceptibility to atherosclerosis has revealed the effects of hemorrheologic factors, among which endothelial shear stress has a prominent role^33^. Thus, miR-21 and -92a are differentially expressed as a function of shear stress. Preferential expression of miR-92a in coronary, but not in carotid lesions that are subjected to low shear stress is in agreement with our *in vivo* data^10^. Recently, our selective analysis of patients with ST-Elevation MI (STEMI) revealed lower levels of miR-92a as well as no regulation of miR-21 in circulating monocytes compared with control patients^12^. Explanations for this discrepancy may lie in a different cellular source of miR-21 and -92a. Other ways low levels of monocytic miR-92a in STEMI patients likely represent a compensatory protective mechanism that might be boosted in response to acute MI.

As newly emerging gene regulators, miRs could be involved in the specific regulation of genes contributing to the development of atherosclerosis^34^. Two of described here atheromiRs-21 and -92 involved in the regulation of vascular performance, could represent targets for the development of new therapeutic strategies against atherosclerosis. Here, we provide strong evidence that miR-92a as an atheromiR, being preferentially expressed in ECs is a potential biomarker for human coronary atherosclerosis.

Several miRs such as miR-92a, which modulate ECs proliferation and inflammation, are up-regulated by disturbed flow in ECs, and contribute to atherosclerosis^31^. By searching miRs target gene predicting database and available published reference, we found 17 potential miR-92a target genes related to vascular inflammation and atherosclerosis (Fig. 2) and only 3 with anti-atherogenic properties, which are RGS3, KLF2 and GDF11 (Table 3). RGS3 inhibits TGF-β1/Smad signalling and may provide protection against pathological changes of adventitial fibroblasts and the development of atherosclerosis^32^. Previous studies showed that the key endothelial transcription factors, KLF2 and KLF4, are direct targets of miR-92a^31^. KLF4 is involved in the regulation of endothelial inflammation through blockade of NF-κB pathway activation, and KLF2 is known to be protective and modulated by flow in ECs^31^. Stimuli such as disturbed flow and oxidized lipids that impose oxidative stress in ECs induce miR-92a, a crucial miRNA that inhibits EC angiogenesis and impairs EC function^35^. SOCS5 has been identified as a novel miR-92a target that is involved in the regulation of endothelial inflammation^36^. SOCSs are key regulators of cytokine-induced responses in hematopoietic as well as nonhematopoietic cells. SOCS5 expression is induced by shear stress and confers anti-inflammatory properties to ECs^37^. At the molecular level, miR-92a targets KLF2, KLF4, and possibly Sirtuin 1 (SIRT1), all of which are tightly associated with redox balance, eNOS-derived NO bioavailability, and the inflammatory state^31^. In regards to endothelial innate immune response, KLF2, KLF4, and SIRT1 suppress the production or antagonize the effect of IL-1β. In terms of translational implications, administration of locked nucleic acid (LNA)-modified antisense miR-92a (LNA-92a) prevents ischemic injury in pigs and ameliorates hyperlipidemia-induced atherosclerosis in mice^38^. However, T cell and macrophage contents in plaques were reduced by antimiR-92a treatment. This was likely because of the antiadhesive effect of miR-92a blockade in ECs, possibly involving KLF2^39^. The expression of miR-92a in the advanced CAP, which usually show a pronounced macrophage content (macrophage marker CD68) reinforce our conclusion that the antiatherogenic effects, which could be observed after miR-92a blockade resulted not only from protection against endothelial dysfunction, but from controlling by both innate and adaptive immunity.

MiR-99a has been associated with the cancer stem cell population in a model of breast- and lung cancer but its role in atherosclerosis remained unknown^40,41^. The present study is the first to describe significantly upregulation of miR-99a in human coronary atherosclerotic plaques versus IMA (p < 0.005, fold changes 3.8). Under the 32 identified miR-99a target genes (see Supplementary Table S3) two are related to atherosclerosis: TRAF7 modulates activity of NF-κB transcription factor, and may thus contribute to pro-atherogenic inflammatory stimulation^42^. The second target gene, FGFR, promotes atherosclerosis development via increased smooth muscle cells (SMCs) proliferation, and by augmenting macrophage accumulation^43^. More research is still needed to verify the pharmacological and diagnostic potential of miR-99a in vascular endothelial cells as well as in atherosclerosis.

In our study we identified three miRs that were down regulated in CAP, namely let-7f, miR-1 and miR-22. Interestingly, for all of them down regulation was already described to be associated with atherosclerosis and in some cases also to be involved in the atherosclerotic process. miR-22 is found in vascular SMCs and ECs. In both cell types, miR-22 is reduced under atherosclerosis: either in human arteries from arteriosclerosis obliterans or in coronary ECs from rats under high fat diet^35,44^. Down regulation of miR-22 in VSMCs enhances proliferation and neointima formation via the cytokine HMGB1. Both processes are known contributors to atherosclerosis. In rats under high fat diet miR-22 is down regulated in the heart which goes along with inflammasome activation. And in coronary ECs antagomirs of miR-22 reduce cell survival and increase expression of pro-inflammatory cytokines. Whether expression of miR-22 in our study is down regulated in ECs or SMCs cannot be decided, as in the plaque material, both cell types can be found. But surely, this decrease in miR-22 levels will create an unfavourable pro-inflammatory situation that promotes plaque formation. Also, miR-1 is found reduced under high fat diet in atherosclerosis prone ApoE knock-out mice, which goes along with enhancement of EC permeability^45^. Interestingly, treatment of these mice with miR-1 attenuated endothelial barrier function. As atherosclerosis often starts with endothelial dysfunction, the reduced miR-1 levels that we detected in plaque material, might have contributed to plaque formation. Finally, also microRNAs of the let-7 family are known to be involved in processes related to atherosclerosis, like cell proliferation, angiogenesis and immuntolerance^46,47^. Interestingly, recently let-7f was found down regulated in plasma probes of poorly controlled diabetic patients that are prone to develop atherosclerotic lesions. 12 months after anti-diabetic therapy, let-7f was normalized^48^. Not only high plasma glucose levels, but also immune activation by LPS or cigarette smoke provokes let-7f decreases^49,50^. The decrease in let-7f levels under cigarette smoke provoked anti-angiogenic effects via modulation TGFbeta-pathway in endothelial cells^50^. Thus, the cellular source of the identified reductions in miRNA levels in atherosclerotic plaques may, primarily, come from ECs and VSMCs, and contribute to atherosclerosis progression.

In conclusion, the miRNA expression profile differs significantly between atherosclerotic plaques and healthy control arteries. The most up-regulated miRs, so-called atheromiRs, are all involved in cellular processes known to be connected with atherosclerosis. Interfering with the miRNA expression in the artery is a potential means to affect the plaque development in many ways, using just a single molecule as the target. Deciphering the complex cell- and context-specific effects of miRs during vascular wound healing appears essential for the development of miRNA-based therapies of atherosclerosis. Furthermore, specific *in vivo* blockade of miR-21 as well as miR-92a expression could reduce vascular inflammation and altered the development of atherosclerosis, decreasing plaque size and promoting a more stable lesion phenotype. MiR-21 and -92a may be a new therapeutic target for proliferative vascular diseases such as atherosclerosis, postangioplasty restenosis, and transplantation vasculopathy.

## Electronic supplementary material

Suppl. Table S1

Suppl. Table S2

Suppl. Table S3

Suppl. Figure S1
