# Supplementary material for: MicroRNA expression profile of human advanced coronary atherosclerotic plaques
Source: Sci Rep. 2018 May 18;8:7823. doi: 10.1038/s41598-018-25690-4 (PMC5959940; doi:10.1038/s41598-018-25690-4)
Supplement: Supplementary file 1 — Suppl. Table S1 [file 41598_2018_25690_MOESM1_ESM.pdf]

MicroRNA expression profile of human advanced coronary atherosclerotic plaques

Mariana Parahuleva[1, 2*], Christoph Lipps[2], Behnoush Parviz[2], Hans Hölschermann[3], Bernhard Schieffer[1], Rainer Schulz[2], Gerhild Euler[2]

Internal Medicine/Cardiology and Angiology, University Hospital of Giessen and Marburg, Location Marburg[1]; Internal Medicine I/Cardiology and Angiology, University Hospital of Giessen and Marburg, Location Giessen[2]; Krankenhaus Bad Homburg Innere Medizin I – Kardiologie, Bad Homburg[3], Germany

Suppl. Table S1. Target genes of the selected miRNAs and their impact on atherosclerosis

miRNA	Target Genes (inter alia)	Source	Impact on Atherosclerosis/Source (inter alia)
miR-1	KCNJ2 (K+ channel subunit Kir2.1)<br>GJA1 (Gap junction alpha-1, connexin 43/Cx43)	Yang et al., 2007, Nat Med.	Foam cell formation (Zhang et al. 2016, J Cell Mol Med.)<br>Neutrophil migration and plaque formation (Wang, H. et al. 2013, Mol Cell Biochem.).
miR-9	PPAR-δ (peroxisome proliferator-activated receptor)-δ	Thulin et al., 2013, Int J Mol Med.	Migration of SMCs (Marx et al. 1998, Circ Res.)
miR-19b	Tumor suppressor 53 (p53)	Fan et al., 2014, RNA	Cell proliferation/apoptosis during plaque formation (Guevara et al. 1999, Nat Med.)
miR-21	Matrix metalloproteinase-9 (MMP-9)	Zhang et al., 2017, Biosci Rep.	Makrophage infiltration, collagen deposition, plaque formation (Wang et al. 2015, Arterioscler Thromb Vasc Biol.; Fan et al. 2014, Exp Mol Pathol.)
miR-22	HMGB1	Huang SC et al. 2017, Cell Physiol Biochem.)	Foam cell formation and plaque formation (Ding et al. 2012, Cell Metab., Huang WQ et al. 2017, Cell Physiol Biochem.)
miR-29b	CX3CL1 (Chemokine C-X3-C Motif Ligand)	Yang et al., 2015, Oral Oncol.	Macrophage infiltration and plaque formation (Teupser et al. 2004, Proc Natl Acad Sci U S A)
miR-92a	KLF2, KLF 4, MYCBP2	Pierre et al., 2017, Eur J Immunol. Shankman et al., 2015, Nat Med.	Inflammation and smooth muscle cell dysfunction (s. Table 3) (Raghuraman et al. 2016, Atherosclerosis.)
miR-99a	TRAF7, Transforming growth factor-β (TGF-β)	Zotti et al., 2012, J Cell Physiol.	Inflammation, chemotaxis, fibrosis, proliferation, and apoptosis (McCaffrey et al. 2010, Front Biosci.; Turcatel et al. 2012, PLoS One)

miR-143	Cyclooxygenase-2 (COX-2)	Hergenreider et al., 2012, Nat Cell Biol.	Regulation of the proinflammatory miR-143 in monocytes of AMI patients (Parahuleva et al. 2017, Sci Rep)
miR-223	Tissue factor (TF)	Li et al., 2014, Atherosclerosis	Plaque formation and thrombosis (Westrick et al. 2001, Circulation)
let-7f	Tissue inhibitor of metalloproteinases 1 (TIMP-1)	Egea et al., 2012, Proc Natl Acad Sci U S A	MMP expression, macrophage infiltration, plaque formation (Rouis et al. 1999, Circulation)
