# Supplementary material for: MicroRNA expression profile of human advanced coronary atherosclerotic plaques
Source: Sci Rep. 2018 May 18;8:7823. doi: 10.1038/s41598-018-25690-4 (PMC5959940; doi:10.1038/s41598-018-25690-4)
Supplement: Supplementary file 2 — Suppl. Table S2 [file 41598_2018_25690_MOESM2_ESM.pdf]

MicroRNA expression profile of human advanced coronary atherosclerotic plaques

Mariana Parahuleva[1, 2*], Christoph Lipps[2], Behnoush Parviz[2], Hans Hölschermann[3], Bernhard Schieffer[1], Rainer Schulz[2], Gerhild Euler[2]

Internal Medicine/Cardiology and Angiology, University Hospital of Giessen and Marburg, Location Marburg[1]; Internal Medicine I/Cardiology and Angiology, University Hospital of Giessen and Marburg, Location Giessen[2]; Krankenhaus Bad Homburg Innere Medizin I – Kardiologie, Bad Homburg[3], Germany

Suppl. Table S2. Predicted target genes of miR-92a found using TargetScan PicTar database.

Gene symbol	Gene Name
MAN2A1	mannosidase, alpha, class 2A, member 1
CPEB3	cytoplasmic polyadenylation element binding protein 3
SLC17A6	solute carrier family 17 (sodium-dependent inorganic phosphate cotransporter), member 6
NFYC	nuclear transcription factor Y, gamma
PIK3R3	phosphoinositide-3-kinase, regulatory subunit 3 (p55, gamma)
HAND1	heart and neural crest derivatives expressed 1
FHL2	four and a half LIM domains 2, transcript variant 1
ZCWCC3	zinc finger, CW type with coiled-coil domain 3
FBXW7	F-box and WD-40 domain protein 7 (archipelago homolog, Drosophila), transcript variant 2
REV3L	REV3-like, catalytic subunit of DNA polymerase zeta (yeast)
FHL2	four and a half LIM domains 2, transcript variant 3
FHL2	four and a half LIM domains 2, transcript variant 4
PCTK1	PCTAIRE protein kinase 1, transcript variant 2
BCL9	B-cell CLL/lymphoma 9
MYO1B	myosin IB
RNF44	ring finger protein 44
RAM2	transcription factor RAM2
BTG2	BTG family, member 2
ARMC1	armadillo repeat containing 1
RAP1B	RAP1B, member of RAS oncogene family
MGC39518	hypothetical protein MGC39518
OAZ3	ornithine decarboxylase antizyme 3
WRNIP1	Werner helicase interacting protein 1, transcript variant 1
NFIA	nuclear factor I/A
FAD104	factor for adipocyte differentiation 104
SSTK	serine/threonine protein kinase SSTK
TSGA14	testis specific, 14

SYN2	synapsin II, transcript variant IIb
LBX1	transcription factor similar to D. melanogaster homeodomain protein lady bird late
G3BP2	Ras-GTPase activating protein SH3 domain-binding protein 2, transcript variant 2
K6IRS2	keratin protein K6irs
CD69	CD69 antigen (p60, early T-cell activation antigen)
SLC9A3R2	solute carrier family 9 (sodium/hydrogen exchanger), isoform 3 regulator 2
BCAT2	branched chain aminotransferase 2, mitochondrial
ING1L	inhibitor of growth family, member 1-like
CLK3	CDC-like kinase 3, transcript variant phclk3
MAP2K4	mitogen-activated protein kinase kinase 4
HPS6	Hermansky-Pudlak syndrome 6
RAB23	member RAS oncogene family, transcript variant 2
GRIA3	glutamate receptor, ionotrophic, AMPA 3, transcript variant flop
FLJ20273	RNA-binding protein
FLJ20399	hypothetical protein FLJ20399
DAG1	dystroglycan 1 (dystrophin-associated glycoprotein 1)
SPHK2	sphingosine kinase 2
C11orf24	chromosome 11 open reading frame 24
LOC283377	hypothetical protein LOC283377
SESN3	sestrin 3
SEMA3A	sema domain, immunoglobulin domain (Ig), short basic domain, secreted, (semaphorin) 3A
TAF15	TAF15 RNA polymerase II, TATA box binding protein (TBP)-associated factor, 68kDa, transcript variant 2
USF2	upstream transcription factor 2, c-fos interacting, transcript variant 1
MEF2D	MADS box transcription enhancer factor 2, polypeptide D (myocyte enhancer factor 2D)
GDF11	growth differentiation factor 11
LIMR	lipocalin-interacting membrane receptor
RSBN1	round spermatid basic protein 1

PPP1R12C	protein phosphatase 1, regulatory (inhibitor) subunit 12C
N-PAC	cytokine-like nuclear factor n-pac
MLR2	ligand-dependent corepressor
SNAPC1	small nuclear RNA activating complex, polypeptide 1, 43kDa
RNF38	ring finger protein 38, transcript variant 1
PDZK3	PDZ domain containing 3, transcript variant 2
KIF5B	kinesin family member 5B
HAND2	heart and neural crest derivatives expressed 2
CIC	capicua homolog (Drosophila)
FHL3	four and a half LIM domains 3
MGLL	monoglyceride lipase, transcript variant 1
CPEB2	cytoplasmic polyadenylation element binding protein 2, transcript variant B
RGS3	regulator of G-protein signalling 3, transcript variant 6
RGS3	regulator of G-protein signalling 3, transcript variant 4
KLF2	Kruppel-like factor 2 (lung)
NOX4	NADPH oxidase 4
LHFPL2	lipoma HMGIC fusion partner-like 2
GIT2	G protein-coupled receptor kinase interactor 2, transcript variant 3
NOMO2	nodal modulator 2, transcript variant 2
NOMO1	nodal modulator 1
NOMO3	nodal modulator 3
SERTAD3	SERTA domain containing 3, transcript variant 1
CDH10	cadherin 10, type 2 (T2-cadherin)
BTBD12	BTB (POZ) domain containing 12
ZDHHC3	zinc finger, DHHC domain containing 3
RPS6KA4	ribosomal protein S6 kinase, 90kDa, polypeptide 4, transcript variant 1
XYLT2	xylosyltransferase II
RBBP6	retinoblastoma binding protein 6, transcript variant 3
NIPBL	Nipped-B homolog (Drosophila), transcript variant A
PLEKHA1	pleckstrin homology domain containing, family A

	(phosphoinositide binding specific) member 1, transcript variant 2
MGC45428	hypothetical protein MGC45428
TACC2	transforming, acidic coiled-coil containing protein 2, transcript variant 2
ZNF287	zinc finger protein 287
ARPC2	actin related protein 2/3 complex, subunit 2, 34kDa, transcript variant 1
TRAF3	TNF receptor-associated factor 3, transcript variant 3
UGP2	UDP-glucose pyrophosphorylase 2, transcript variant 2
H3F3B	H3 histone, family 3B (H3.3B)
NSMAF	neutral sphingomyelinase (N-SMase) activation associated factor
PCDH11Y	protocadherin 11 Y-linked, transcript variant c
PCDH11X	protocadherin 11 X-linked, transcript variant c
SIM2	single-minded homolog 2 (Drosophila), transcript variant SIM2
NELF	nasal embryonic LHRH factor
SLC32A1	solute carrier family 32 (GABA vesicular transporter), member 1
RFX1	regulatory factor X, 1 (influences HLA class II expression)
ITGAV	integrin, alpha V (vitronectin receptor, alpha polypeptide, antigen CD51)
ITGA5	integrin, alpha 5 (fibronectin receptor, alpha polypeptide)
HHIP	hedgehog interacting protein
LATS2	LATS, large tumor suppressor, homolog 2 (Drosophila)
TCF21	transcription factor 21, transcript variant 2
KIAA0063	KIAA0063 gene product
KCNK10	potassium channel, subfamily K, member 10, transcript variant 3
FLJ22313	hypothetical protein FLJ22313
SNX2	sorting nexin 2
ABCG4	ATP-binding cassette, sub-family G (WHITE), member 4
TGIF	TGFB-induced factor (TALE family homeobox), transcript variant 8
GRK5	G protein-coupled receptor kinase 5
NEF3	neurofilament 3 (150kDa medium)
ADM	adrenomedullin

TCF2	transcription factor 2, hepatic; LF-B3; variant hepatic nuclear factor, transcript variant a
ADCY3	adenylate cyclase 3
KIAA1409	KIAA1409
DSCAML1	Down syndrome cell adhesion molecule like 1
DAB2IP	DAB2 interacting protein
TFCP2L2	transcription factor CP2-like 2, transcript variant 1
RBM9	RNA binding motif protein 9
MGC11061	hypothetical protein MGC11061
NRF1	nuclear respiratory factor 1
IRS2	insulin receptor substrate 2
ARHGEF17	Rho guanine nucleotide exchange factor (GEF) 17
SLC6A1	solute carrier family 6 (neurotransmitter transporter, GABA), member 1
GFPT2	glutamine-fructose-6-phosphate transaminase 2
FNBP4	formin binding protein 4
MIDORI	likely ortholog of mouse myocytic induction/differentiation originator
WWP2	WW domain containing E3 ubiquitin protein ligase 2, transcript variant 1
MYO18A	myosin XVIIIA, transcript variant 1
FLJ21616	hypothetical protein FLJ21616
FLJ11838	hypothetical protein FLJ11838
ADAM19	a disintegrin and metalloproteinase domain 19 (meltrin beta), transcript variant 2
PRDM13	PR domain containing 13
CPEB4	cytoplasmic polyadenylation element binding protein 4
SRPR	signal recognition particle receptor ('docking protein')
NPC1	Niemann-Pick disease, type C1
VPS54	vacuolar protein sorting 54 (yeast), transcript variant 2, mRNA
KLF4	Kruppel-like factor 4 (gut)
ARF1	ADP-ribosylation factor 1
FUNDC1	FUN14 domain containing 1

GOLGA4	golgi autoantigen, golgin subfamily a, 4
FOXG1B	forkhead box G1B
NFIB	nuclear factor I/B
C22orf5	chromosome 22 open reading frame 5
CLK3	CDC-like kinase 3, transcript variant phclk3/152
PCANAP6	prostate cancer associated protein 6
CEBPA	CCAAT/enhancer binding protein (C/EBP), alpha
CXXC5	CXXC finger 5
MARK1	MAP/microtubule affinity-regulating kinase 1
SUI1	putative translation initiation factor
TNFSF6	tumor necrosis factor (ligand) superfamily, member 6
C20orf39	chromosome 20 open reading frame 39
SEC24C	SEC24 related gene family, member C (S. cerevisiae), transcript variant 1
SDFR1	stromal cell derived factor receptor 1, transcript variant beta
C9orf150	chromosome 9 open reading frame 150
FLJ46347	hypothetical LOC389064
HERC2	hect domain and RLD 2
HNRPA1	heterogeneous nuclear ribonucleoprotein A1, transcript variant 1
KIAA1196	KIAA1196 protein
DUSP5	dual specificity phosphatase 5
MLL5	myeloid/lymphoid or mixed-lineage leukemia 5 (trithorax homolog, Drosophila)
ZDHHC5	zinc finger, DHHC domain containing 5
TRAM2	translocation associated membrane protein 2
KIAA1043	KIAA1043 protein
ALS2CR3	amyotrophic lateral sclerosis 2 (juvenile) chromosome region, candidate 3
13CDNA73	hypothetical protein CG003
ATP2A2	ATPase, Ca++ transporting, cardiac muscle, slow twitch 2, transcript variant 2
GPR124	G protein-coupled receptor 124
HAPIP	huntingtin-associated protein interacting protein (duo)

C6orf62	chromosome 6 open reading frame 62
MYCBP2	MYC binding protein 2
MGC29816	hypothetical protein MGC29816
SLC38A2	solute carrier family 38, member 2
ZFHX1B	zinc finger homeobox 1b
COL12A1	collagen, type XII, alpha 1, transcript variant long
FLJ10159	hypothetical protein FLJ10159
EGR2	early growth response 2 (Krox-20 homolog, Drosophila)
FLJ13855	hypothetical protein FLJ13855
RYR3	ryanodine receptor 3
CREB1	cAMP responsive element binding protein 1, transcript variant A
SP192	hypothetical protein SP192
RANBP9	RAN binding protein 9
FBN1	fibrillin 1 (Marfan syndrome)
COL1A2	collagen, type I, alpha 2
GATA6	GATA binding protein 6
PTPRO	protein tyrosine phosphatase, receptor type, O, transcript variant 2
CACNA1C	calcium channel, voltage-dependent, L type, alpha 1C subunit
MGC39325	hypothetical protein MGC39325
SLC24A3	solute carrier family 24 (sodium/potassium/calcium exchanger), member 3
DNAJB12	DnaJ (Hsp40) homolog, subfamily B, member 12, transcript variant 2
NFAT5	nuclear factor of activated T-cells 5, tonicity-responsive, transcript variant 3
MLL5	myeloid/lymphoid or mixed-lineage leukemia 5 (trithorax homolog, Drosophila)
CBFA2T3	core-binding factor, runt domain, alpha subunit 2; translocated to, 3, transcript variant 2
SUV420H1	suppressor of variegation 4-20 homolog 1 (Drosophila), transcript variant 1
CBLN4	cerebellin 4 precursor
DYRK2	dual-specificity tyrosine-(Y)-phosphorylation regulated kinase 2, transcript variant 1

SCUBE3	signal peptide, CUB domain, EGF-like 3
POLK	polymerase (DNA directed) kappa
PHF15	PHD finger protein 15
FLJ11011	hypothetical protein FLJ1101, transcript variant 3
DUSP10	dual specificity phosphatase 10, transcript variant 2
DUSP6	dual specificity phosphatase 6, transcript variant 1
JUN	v-jun sarcoma virus 17 oncogene homolog (avian)
TAFA1	TAFA1 protein
STRN	striatin, calmodulin binding protein
ELOVL4	elongation of very long chain fatty acids (FEN1/Elo2, SUR4/Elo3, yeast)-like 4
ZFPM2	zinc finger protein, multitype 2
DSC2	desmocollin 2, transcript variant Dsc2a
SSFA2	sperm specific antigen 2
USP28	ubiquitin specific protease 28
NS3TP2	HCV NS3-transactivated protein 2
POLS	polymerase (DNA directed) sigma
GOLGA3	golgi autoantigen, golgin subfamily a, 3
DNAJB12	DnaJ (Hsp40) homolog, subfamily B, member 12, transcript variant 1
EPS8	epidermal growth factor receptor pathway substrate 8
DPP10	dipeptidylpeptidase 10, transcript variant 1
DKFZP566B183	DKFZP566B183 protein
TEF	thyrotrophic embryonic factor
KCNJ3	potassium inwardly-rectifying channel, subfamily J, member 3
INSIG1	insulin induced gene 1, transcript variant 2
PAX3	paired box gene 3 (Waardenburg syndrome 1), transcript variant PAX3E
PAX3E	
TOP1	topoisomerase (DNA)
EPC2	enhancer of polycomb homolog 2 (Drosophila)
MYLIP	myosin regulatory light chain interacting protein
RNF4	ring finger protein 4

HMGA2	high mobility group AT-hook 2
PAX3 PAX3H	paired box gene 3 (Waardenburg syndrome 1), transcript variant PAX3H
DSC2	desmocollin 2, transcript variant Dsc2b
SNN	stannin
NEGR1	neuronal growth regulator 1
INSIG1	insulin induced gene 1, transcript variant 1
PAX3	paired box gene 3 (Waardenburg syndrome 1), transcript variant PAX3
SCN3A	sodium channel, voltage-gated, type III, alpha
INSIG1	insulin induced gene 1, transcript variant 3
BAZ2B	bromodomain adjacent to zinc finger domain, 2B
CIT	citron (rho-interacting, serine/threonine kinase 21)
P66beta	transcription repressor p66 beta component of the MeCP1 complex
PPP1R12A	protein phosphatase 1, regulatory (inhibitor) subunit 12A
EN2	engrailed homolog 2
PTGER4	prostaglandin E receptor 4 (subtype EP4)
FLJ38101	hypothetical protein FLJ38101
PER2	period homolog 2 (Drosophila), transcript variant 1
SDC2	syndecan 2 (heparan sulfate proteoglycan 1, cell surface-associated, fibroglycan)
DLGAP2	discs, large (Drosophila) homolog-associated protein 2
ITR	intimal thickness-related receptor
PAX3 PAX3D	paired box gene 3 (Waardenburg syndrome 1), transcript variant PAX3D
UBE2G1	ubiquitin-conjugating enzyme E2G 1 (UBC7 homolog, C. elegans), transcript variant 1
PITPNA	phosphatidylinositol transfer protein, alpha
HAS3	hyaluronan synthase 3, transcript variant 1
CNTN4	contactin 4, transcript variant 1
RAD21	RAD21 homolog (S. pombe)
NPTX1	neuronal pentraxin I

BAZ2A	bromodomain adjacent to zinc finger domain, 2A
UBE2G1	ubiquitin-conjugating enzyme E2G 1 (UBC7 homolog, C. elegans), transcript variant 2
LKAP	limkain b1, transcript variant 1
FLJ11011	hypothetical protein FLJ11011, transcript variant 1
OACT2	O-acyltransferase (membrane bound) domain containing 2
NLK	nemo like kinase
C13orf6	chromosome 13 open reading frame 6
BCL2L11	BCL2-like 11 (apoptosis facilitator), transcript variant 6
CAMKK2	calcium/calmodulin-dependent protein kinase kinase 2, beta, transcript variant 7
CAMKK2	calcium/calmodulin-dependent protein kinase kinase 2, beta, transcript variant 1
ADAMTSL3	ADAMTS-like 3
KLF12	Kruppel-like factor 12, transcript variant 1
PDE4D	phosphodiesterase 4D, cAMP-specific (phosphodiesterase E3 dunce homolog, Drosophila)
LIN28	lin-28 homolog (C. elegans)
BCL2L11	BCL2-like 11 (apoptosis facilitator), transcript variant 7
FBXO33	F-box protein 33
SGPP1	sphingosine-1-phosphate phosphatase 1
NR4A3	nuclear receptor subfamily 4, group A, member 3, transcript variant 1
PAPOLA	poly(A) polymerase alpha
OSR1	oxidative-stress responsive 1
PCAF	p300/CBP-associated factor
ZDHHC21	zinc finger, DHHC domain containing 21
FMR1	fragile X mental retardation 1
LUZP1	leucine zipper protein 1
STAG2	stromal antigen 2
DMXL1	Dmx-like 1
TEAD1	TEA domain family member 1 (SV40 transcriptional enhancer factor)
SYNJ1	synaptojanin 1, transcript variant 1

PRKCE	protein kinase C, epsilon
E2F3	E2F transcription factor 3
PAPOLB	poly(A) polymerase beta (testis specific)
C17orf39	chromosome 17 open reading frame 39
RAB14	RAB14, member RAS oncogene family
CFL2	cofilin 2 (muscle), transcript variant 1
TSC1	tuberous sclerosis 1
ARRDC4	arrestin domain containing 4
MCL1	myeloid cell leukemia sequence 1 (BCL2-related), transcript variant 1
PAFAH1B1	platelet-activating factor acetylhydrolase, isoform Ib, alpha subunit 45kDa
ESRRG	estrogen-related receptor gamma, transcript variant 1
RNF3	ring finger protein 3
SATB2	SATB family member 2
ATRX	alpha thalassemia/mental retardation syndrome X-linked (RAD54 homolog, S. cerevisiae), transcript variant 1
ARRDC3	arrestin domain containing 3
SLC12A2	solute carrier family 12 (sodium/potassium/chloride transporters), member 2
TBDN100	transcriptional coactivator tubedown-100, transcript variant 1
APRIN	androgen-induced proliferation inhibitor
BCL11A	B-cell CLL/lymphoma 11A (zinc finger protein), transcript variant 1
HSHIN1	HIV-1 induced protein HIN-1, transcript variant 1
GPR85	G protein-coupled receptor 85
CHES1	checkpoint suppressor 1
CUL3	cullin 3
SEC10L1	SEC10-like 1 (S. cerevisiae)
QKI	quaking homolog, KH domain RNA binding (mouse)
transcript variant 2	
OGT	O-linked N-acetylglucosamine (GlcNAc) transferase (UDP-N-acetylglucosamine: polypeptide-N-acetylglucosaminyl transferase), transcript variant 3

QKI transcript variant 3	quaking homolog, KH domain RNA binding (mouse)
QKI transcript variant 4	quaking homolog, KH domain RNA binding (mouse)
