# Supplementary material for: MicroRNA expression profile of human advanced coronary atherosclerotic plaques
Source: Sci Rep. 2018 May 18;8:7823. doi: 10.1038/s41598-018-25690-4 (PMC5959940; doi:10.1038/s41598-018-25690-4)
Supplement: Supplementary file 3 — Suppl. Table S3 [file 41598_2018_25690_MOESM3_ESM.pdf]

MicroRNA expression profile of human advanced coronary atherosclerotic plaques

Mariana Parahuleva[1, 2*], Christoph Lipps[2], Behnoush Parviz[2], Hans Hölschermann[3], Bernhard Schieffer[1], Rainer Schulz[2], Gerhild Euler[2]

Internal Medicine/Cardiology and Angiology, University Hospital of Giessen and Marburg, Location Marburg[1]; Internal Medicine I/Cardiology and Angiology, University Hospital of Giessen and Marburg, Location Giessen[2]; Krankenhaus Bad Homburg Innere Medizin I – Kardiologie, Bad Homburg[3], Germany

Suppl. Table S3. Predicted target genes of miR-99a found using TargetScan PicTar database.

Gene symbol	Gene Name
SMARCA5	SWI/SNF related, matrix associated, actin dependent regulator of chromatin, subfamily a, member 5
BAZ2A	bromodomain adjacent to zinc finger domain, 2A
VLDLR	very low density lipoprotein receptor
HS3ST3B1	heparan sulfate (glucosamine) 3-O-sulfotransferase 3B1
HS3ST2	heparan sulfate (glucosamine) 3-O-sulfotransferase 2
FOXA1	forkhead box A1
EPC2	enhancer of polycomb homolog 2 (Drosophila)
FRAP1	FK506 binding protein 12-rapamycin associated protein 1
EIF2C2	eukaryotic translation initiation factor 2C, 2
C4orf16	chromosome 4 open reading frame 16
FZD8	frizzled homolog 8 (Drosophila)
INSM1	insulinoma-associated 1
HOXA1	homeo box A1, transcript variant 1
KIAA1579	hypothetical protein FLJ10770
CDW92	CDW92 antigen
MGC39518	hypothetical protein MGC39518
HOXA1	homeo box A1, transcript variant 2
MBNL1	muscleblind-like (Drosophila), transcript variant 1
MBNL1	muscleblind-like (Drosophila), transcript variant 7
PHOX2B	paired-like homeobox 2b
TA-KRP	T-cell activation kelch repeat protein
FGFR3	fibroblast growth factor receptor 3 (achondroplasia, thanatophoric dwarfism), transcript variant 1
TRIB2	tribbles homolog 2 (Drosophila)
ZDHHC18	zinc finger, DHHC domain containing 18
ZZEF1	zinc finger, ZZ type with EF hand domain 1
MTMR3	myotubularin related protein 3, transcript variant 3
ICMT	isoprenylcysteine carboxyl methyltransferase, transcript variant 1
OGT	O-linked N-acetylglucosamine (GlcNAc) transferase (UDP-N-acetylglucosamine:polypeptide-N-acetylglucosaminyl

	transferase: polypeptide-N-acetylglucosaminyl transferase) (OGT), transcript variant 3
TRAF7	TNF receptor-associated factor 7, transcript variant 2
CYP26B1	cytochrome P450, family 26, subfamily B, polypeptide 1
CTDSPL	CTD (carboxy-terminal domain, RNA polymerase II, polypeptide A) small phosphatase-like
ADCY1	adenylate cyclase 1 (brain)
