# Supplementary material for: MicroRNA expression profile of human advanced coronary atherosclerotic plaques
Source: Sci Rep. 2018 May 18;8:7823. doi: 10.1038/s41598-018-25690-4 (PMC5959940; doi:10.1038/s41598-018-25690-4)
Supplement: Supplementary file 4 — Suppl. Figure S1 [file 41598_2018_25690_MOESM4_ESM.pdf]

MicroRNA expression profile of human advanced coronary atherosclerotic plaques

Mariana Parahuleva[1,2*], Christoph Lipps[2], Behnoush Parviz[2], Hans Hölschermann[3], Bernhard Schieffer[1], Rainer Schulz[2], Gerhild Euler[2]

Internal Medicine/Cardiology and Angiology, University Hospital of Giessen and Marburg, Location Marburg[1]; Internal Medicine I/Cardiology and Angiology, University Hospital of Giessen and Marburg, Location Giessen[2]; Krankenhaus Bad Homburg Innere Medizin I – Kardiologie, Bad Homburg[3], Germany

Study design flow chart

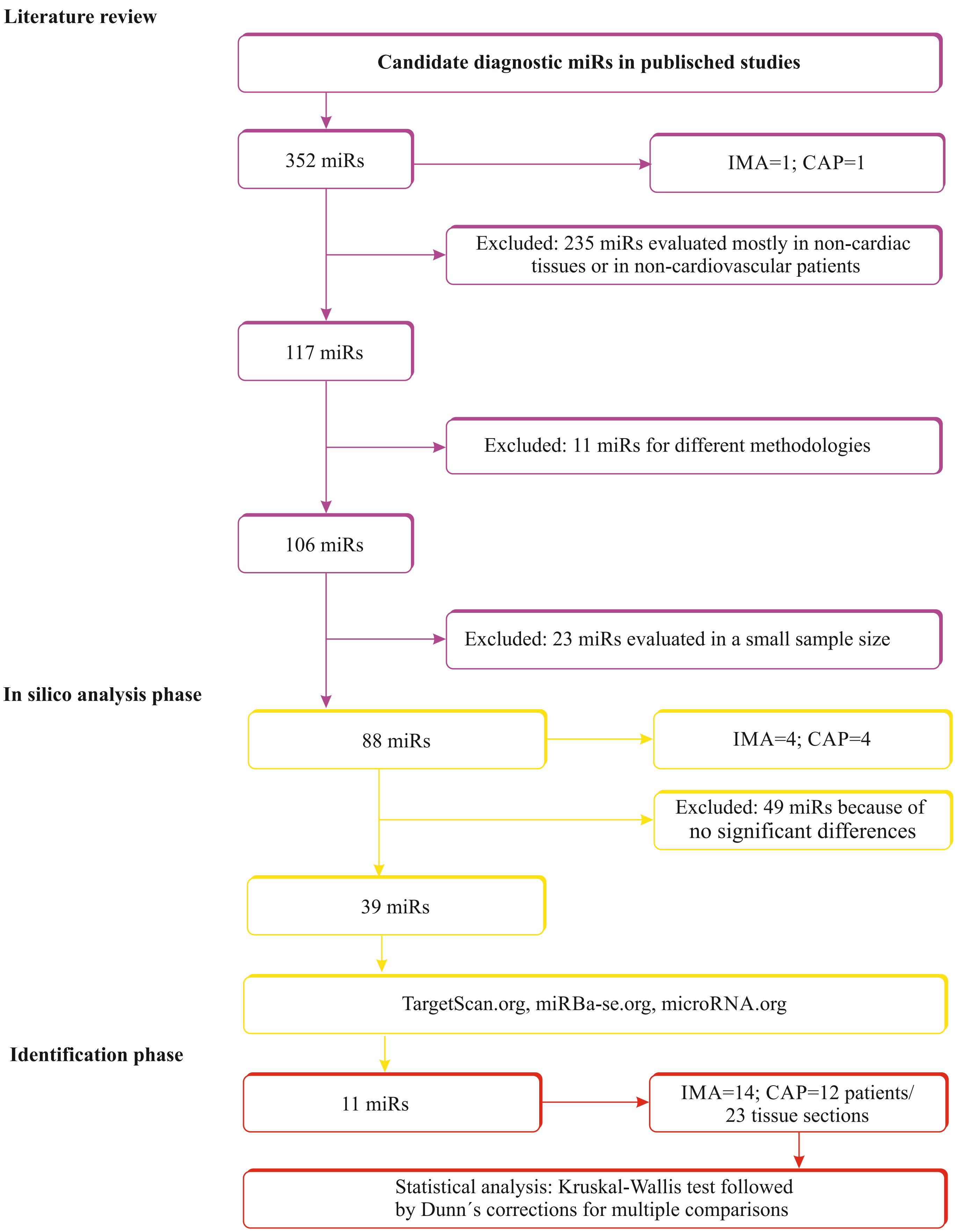

miRs, miRNAs; CAP, coronary artery plaques; IMA, internal mammary arteries
